# Empirical Antibiotic Activity and Outcomes in Pediatric Gram-negative Bloodstream Infections: A Rank-based Composite Outcome Analysis

**DOI:** 10.1093/cid/ciag234

**Published:** 2026-04-09

**Authors:** Sophie C H Wen, Melissa J Hardy, Abdullah T Aslan, Patrick N A Harris, Mark D Chatfield, Colleen L Lau, Geoffrey Spurling, Julia E Clark, Sonia Dougherty, Christopher C Blyth, Philip N Britton, Vanessa Clifford, Gabrielle M Haeusler, Brendan McMullan, Ushma Wadia, Luregn Schlapbach, Henry Chambers, David L Paterson, Adam D Irwin, the PAEDS Network, Jeremy Carr, Nan Vasilunas, Joshua R Francis, Kristine Macartney, Nigel W Crawford, Nicholas Wood, Jim Buttery, Helen Marshall, Elizabeth Elliott, Peter Richmond, Emma Carey, Alissa McMinn, Karen Bellamy, Guillian Hunter, Kathryn Meredith, Laura Rost, Nicole Kerly, Sara Cook, Natasha Doran, Laura Francis, Christine Heath, Carolyn Finucane, Mehyar Khair Baik, Laura Lopez, Catherine Glover

**Affiliations:** The University of Queensland, Centre for Clinical Research, Brisbane, Queensland, Australia; Infection Management Prevention Service, Children's Health Queensland, Brisbane, Queensland, Australia; The University of Queensland, Centre for Clinical Research, Brisbane, Queensland, Australia; The University of Queensland, Centre for Clinical Research, Brisbane, Queensland, Australia; Sunshine Coast University Hospital, Department of General Medicine, Sunshine Coast, Queensland, Australia; The University of Queensland, Centre for Clinical Research, Brisbane, Queensland, Australia; The University of Queensland, Centre for Clinical Research, Brisbane, Queensland, Australia; The University of Queensland, Centre for Clinical Research, Brisbane, Queensland, Australia; The University of Queensland, General Practice Clinical Unit, Brisbane, Queensland, Australia; Infection Management Prevention Service, Children's Health Queensland, Brisbane, Queensland, Australia; Infection Management Prevention Service, Children's Health Queensland, Brisbane, Queensland, Australia; Wesfarmer's Centre of Vaccines and Infectious Diseases, Telethon Kids Institute, Perth, Western Australia, Australia; Department of Infectious Diseases, Perth Children's Hospital, Perth, Western Australia, Australia; Department of Microbiology, PathWest Laboratory Network, Perth, Western Australia, Australia; School of Medicine, University of Western Australia, Perth, Western Australia, Australia; Department of Infectious Diseases and Microbiology, The Children's Hospital Westmead, Sydney, New South Wales, Australia; Sydney Medical School and Sydney Infectious Diseases, University of Sydney, Sydney, New South Wales, Australia; Infectious Diseases Department, Royal Children's Hospital, Melbourne, Victoria, Australia; Clinical Infections Group, Murdoch Children's Research Institute, Melbourne, Victoria, Australia; Infectious Diseases Department, Royal Children's Hospital, Melbourne, Victoria, Australia; Clinical Infections Group, Murdoch Children's Research Institute, Melbourne, Victoria, Australia; Infectious Diseases and Microbiology, Sydney Children's Hospital, Sydney, New South Wales, Australia; Faculty of Medicine and Health, University of New South Wales, Sydney, New South Wales, Australia; Wesfarmer's Centre of Vaccines and Infectious Diseases, Telethon Kids Institute, Perth, Western Australia, Australia; Department of Infectious Diseases, Perth Children's Hospital, Perth, Western Australia, Australia; School of Medicine, University of Western Australia, Perth, Western Australia, Australia; Department of Intensive Care and Neonatology, and Children's Research Center, University Children's Hospital Zurich, University of Zurich, Zurich, Switzerland; Child Health Research Centre, University of Queensland, Brisbane, Queensland, Australia; University of California SanFrancisco, San Francisco, USA; ADVANCE-ID, Saw Swee Hock School of Public Health, National University of Singapore, Singapore; Infectious Diseases Translational Research Programme, Yong Loo Lin School of Medicine, National University of Singapore, Singapore; The University of Queensland, Centre for Clinical Research, Brisbane, Queensland, Australia; Infection Management Prevention Service, Children's Health Queensland, Brisbane, Queensland, Australia

**Keywords:** Gram-negative, pediatric, bloodstream infections, antimicrobial therapy, outcome

## Abstract

**Background:**

Timely active empirical therapy (AET) may improve outcomes following bloodstream infection. In high-income settings, low pediatric mortality following gram-negative bloodstream infection (GNBSI) limits its value as a sole endpoint. Composite measures that incorporate multiple clinically relevant outcomes may detect differences more readily. We assessed the effect of AET on clinical outcomes in pediatric GNBSI.

**Methods:**

We analyzed data from a prospective surveillance study of hospitalized Australian children with GNBSI. A ranked composite endpoint included 30-day all-cause mortality (30D ACM), time to death, intensive care unit (ICU) admission, relapse, and hospitalization duration. Baseline differences were adjusted using inverse probability of treatment weighting. Generalized pairwise comparison (GPC) generated win statistics. Analyses were stratified by comorbidity, acquisition setting, and organism.

**Results:**

Active empirical therapy occurred in 597 episodes and inactive empirical therapy (IET) in 62. Median treatment duration was similar (AET 10 vs IET 11 days, *P* = .44). Generalized pairwise comparison analysis showed an adjusted win ratio of 1.04 (95% CI .72–1.52, *P* = .83), indicating no significant difference. Contributions from each composite component (AET vs IET) were the following: 30D ACM (2.2% vs 1.3%), time to death (0.02% vs 0.01%), ICU admission (7.5% vs 9.1%), relapse (2.6% vs 1.4%), and hospitalization duration (37.3% vs 36.0%). Stratified estimates were similar for comorbidity and acquisition.

**Conclusions:**

In this large multicenter pediatric GNBSI cohort, AET was not associated with improved outcomes. In high-resource settings, the impact of initial antibiotic activity may be smaller than traditionally assumed, though timely effective therapy remains essential for severe illness and low-resource settings.

## Teaser

In this multicenter pediatric GNBSI cohort, active empirical therapy (AET) was not associated with improved outcomes. The impact of AET in high-resource settings may be smaller than assumed, though timely effective therapy remains critical for severe illness and low-resource settings.

## Graphical Abstract

**Graphical Abstract:**
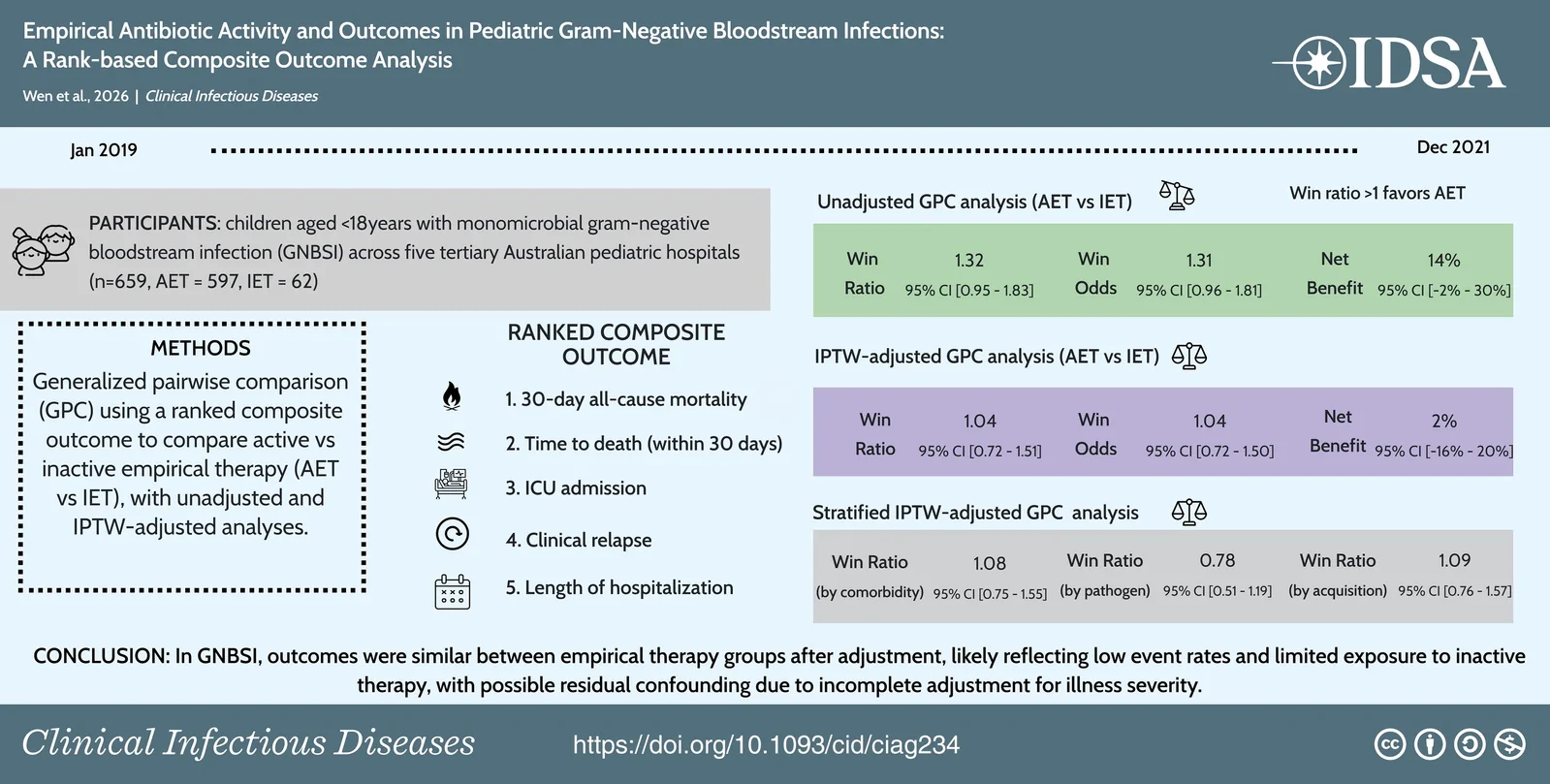
This graphical abstract is also available at Tidbit: https://tidbitapp.io/institutional-portal/clinical-infectious-diseases/tidbits/empirical-antibiotic-activity-and-outcomes-in-pediatric-gram-negative-bloodstream-infections-a-rank-based-composite-outcome-analysis/update

Gram-negative bloodstream infections (GNBSIs) are a major cause of morbidity and mortality in children, particularly those immunocompromised or critically ill [1]. In many settings, gram-negative organisms predominate among pediatric BSIs [2–4]. These infections often progress rapidly, requiring empirical antibiotics before microbiological confirmation, and rising antimicrobial resistance has increased pressure to broaden coverage to avoid delays in effective treatment [5].

Delayed active antibiotic therapy has been linked with adverse outcomes in Gram-negative infections; although this may reflect confounding by antimicrobial resistance or illness severity [6, 7]. Early in vitro active empirical therapy (AET) has been associated with improved survival and shorter hospital stay, particularly in adults, whereas inactive empirical therapy (IET) may lead to therapeutic failure and increased mortality [7–9].

In children, evidence remains limited and largely retrospective [5, 6, 10, 11]. A global cohort study of neonatal and pediatric BSIs reported higher 30-day mortality with IET [5], whereas a Dutch multicenter study of extended spectrum beta-lactamase (ESBL) producing Enterobacterales BSI, including few children (10%), found no association between early AET and 30-day mortality (adjusted odds ratio of 1.53, 95% CI .68–3.45) [12].

All-cause mortality is often the primary outcome in BSI trials. However, mortality following GNBSI in children,particularly in high-resource settings, is relatively low, making differences in mortality difficult to detect [1, 3]. Alternative outcomes, such as intensive care unit (ICU) admission or hospitalization duration, may better capture disease burden, requiring analytical methods that integrate multiple clinically relevant outcomes.

Generalized pairwise comparison (GPC) is a rank-based method that prioritizes outcomes by clinical relevance [13], comparing individuals across a predefined outcome hierarchy to estimate win statistics accounting for direction and magnitude of differences. Although increasingly explored in infectious diseases research [14], GPC has not been applied to pediatric BSIs. It clarifies how individual outcomes contribute to overall treatment effects, extending assessment beyond mortality alone. However, active empirical coverage cannot feasibly or ethically be tested in a randomized controlled trial; observational studies therefore remain the only practical approach.

This study aimed to apply GPC to an observational multicenter dataset to evaluate the impact of AET on clinical outcomes in pediatric GNBSI, using a ranked composite endpoint that reflects clinical priorities.

## METHODS

### Study Design

Data from a prospective surveillance study of GNBSI in hospitalized Australian children through the national Pediatric Active Enhanced Disease Surveillance (PAEDS) network (www.paeds.org.au) [15] between January 2019 and December 2021 were utilized [1]. Full methodology can be found elsewhere [1]. Briefly, children aged <18 years with GNBSI were recruited from 5 Australian tertiary pediatric hospitals. Demographics, comorbidities, infection source, organisms and antimicrobial susceptibilities, treatment, and clinical outcomes were collected. Only monomicrobial GNBSIs were included.

### Definitions

The timing of both blood culture collection and antibiotic administration was recorded by calendar date rather than exact time. To minimize misclassification due to calendar-day recording, AET was defined as any parenteral antibiotics given within 3 days of blood culture sampling, which had in vitro activity against the index Gram-negative bacteria. Episodes not meeting this definition were classified as IET. Targeted therapy was defined as therapy administered after the 3-calendar-day window. Oral antibiotics were not considered as this conflicts with recommended pediatric clinical practice [16]. Minimum inhibitory concentrations (MICs) were interpreted using European Committee on Antimicrobial Susceptibility Testing (EUCAST) clinical breakpoints v13.0 2023 [17]. For this study, aminoglycoside monotherapy meeting EUCAST susceptibility criteria was considered AET, despite current guidance recommending aminoglycosides for systemic infections being used in combination with another active agent [18]. Organisms were excluded where no EUCAST clinical breakpoints were available [17]. In Enterobacterales, third-generation cephalosporin (3GC) resistance (ceftriaxone MIC >2 mg/L or ceftazidime >4 mg/L) was used to indicate the presence of either ESBL or other cephalosporinases (eg AmpC). For organisms that are deemed at moderate risk of clinically significant Amp-C production (*Enterobacter cloacae complex*, *Klebsiella aerogenes*, and *Citrobacter freundii*), 3GC and piperacillin-tazobactam were considered inactive therapy regardless of MIC [19]. Cefepime and carbapenems were considered active if MICs were ≤4 mg/L and ≤8 mg/L (meropenem)/≤4 mg/L (imipenem), respectively. Organisms that are considered low risk for Amp-C production (*Serratia* spp, *Morganella* spp, *Providencia* spp and *C. koseri*), 3GC and piperacillin/tazobactam were considered active if MICs were in range [19]. Acquisition was classified as non–healthcare associated if BSI onset occurred <48 hours after hospital admission, with no central venous catheter (CVC) in place, and no total parenteral nutrition or surgery within the preceding 30 days. BSI onset occurring ≥48 hours after hospital admission was classified as hospital-onset infection. GNBSI with onset in the ICU were included within this hospital-onset category. All deaths were clinically assessed locally to determine whether GNBSI was contributory. All other clinical definitions can be found elsewhere [1].

### Ranked Composite Endpoint

Candidate endpoints, identified from available data (Supplementary Materials 1), underwent review by the PAEDS clinician group together with a parent representative providing insights grounded in lived experience caring for a child with BSI. Final selection and hierarchy were determined by majority agreement. The ranked composite endpoint comprised: (1) 30-day all-cause mortality (ACM), (2) time to death within 30 days, (3) ICU admission, (4) clinical relapse, and (5) duration of hospitalization from BSI onset (censored at 90 days). For patients who died beyond 30 days, the time to death (censored at 90 days) was used in place of duration of hospitalization.

### GPC Approach and Inverse Probability of Treatment Weighting

**Figure 1.**
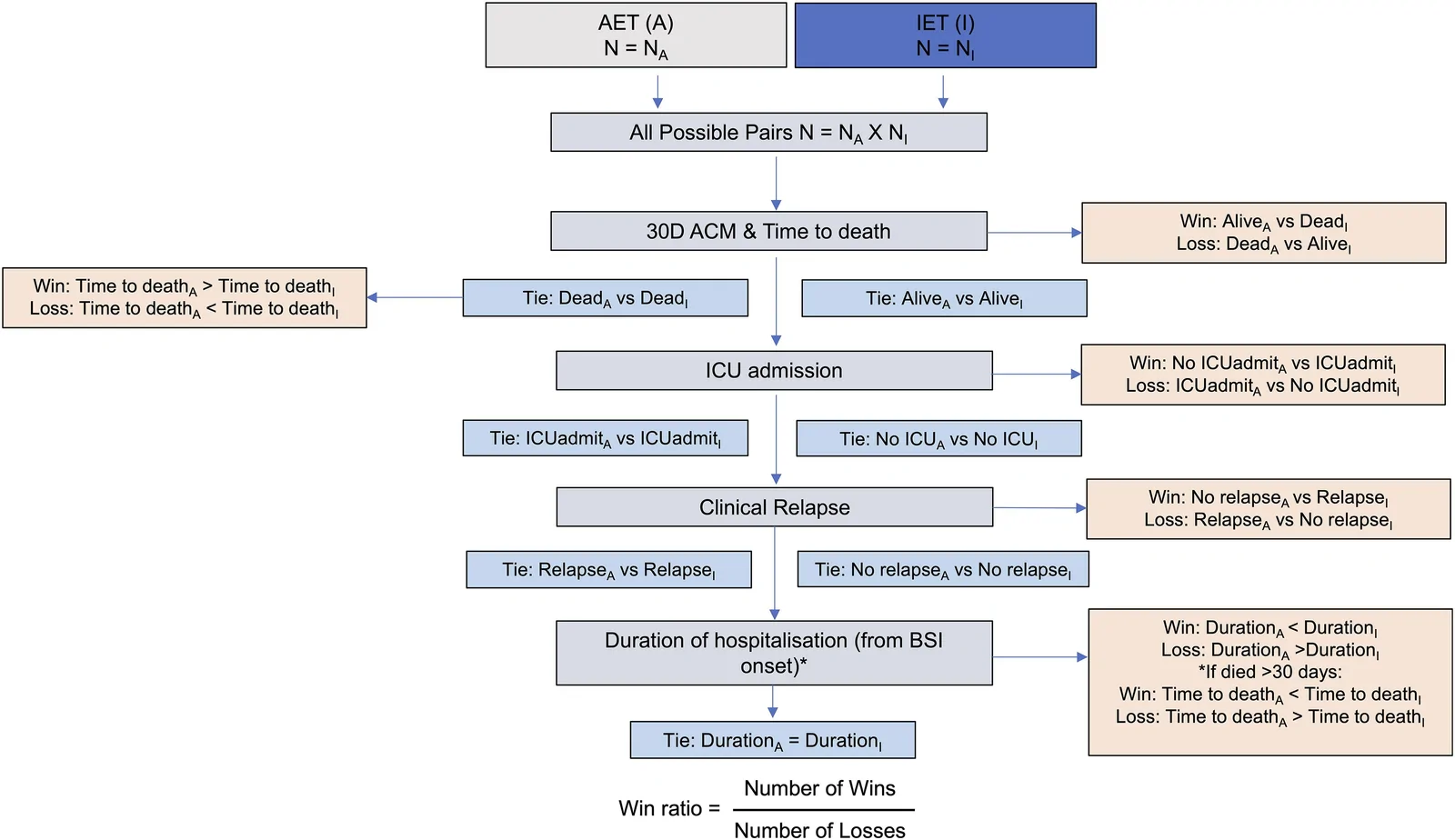
Generalized pairwise comparison approach with a ranked composite endpoint, prioritizing time to all-cause mortality before intensive care unit admission, clinical relapse and duration of hospitalization. Every patient in the active empirical therapy group (*A*) is compared with every patient in the inactive empirical therapy group (*I*) for each endpoint and in the event of a tie, it moves onto the next outcome in the hierarchy.Abbreviations: 30D, 30 d; AET, active empirical therapy; ACM, all-cause mortality; BSI, bloodstream infection; ICU, intensive care unit; IET, inactive empirical therapy; NB, probability (AET better)—probability (IET better); WO, (wins + ½ ties) ÷ (losses + ½ ties); WR, wins ÷ losses.

Generalized pairwise comparison analyses using the unmatched approach were conducted, in which each patient in the AET group was compared with each patient in the IET group. Comparisons proceeded down a predefined outcome hierarchy, beginning with the most clinically important endpoint until a win, loss, or tie was determined. Patients in the AET group were assigned a “win” or “loss” depending on which individual in the pair experienced the outcome first. If neither or both experienced the outcome, the result was considered a tie (Figure 1). From these comparisons, the win ratio (WR) was calculated as wins for AET divided by losses. A WR of 1 indicated no difference between groups, while values >1 favored AET and values <1 favored IET. To account for ties, win odds were estimated, adjusting the WR by allocating half of the tied comparisons to each group. Net benefit was calculated as the difference between the probability that a randomly selected patient in the AET group has a better outcome than one in the IET group and the reverse. It ranges from −1 to 1, where zero indicates no treatment difference, positive values favor AET, and negative values favor IET. Baseline characteristic imbalances were addressed using inverse probability of treatment weighting (IPTW), where patients are weighted by the inverse probability of receiving the treatment they actually received, creating a pseudo-population with balanced covariates. Covariates for adjustment were selected a priori (Table 1) drawing on our previous work in pediatric GNBSI [1] and informed by approaches used in the Eurobact2 study [9] and the target trial emulation by Tingsgård et al [20]. Further details are provided in Supplementary Materials (2). Generalized pairwise comparison analyses were conducted on unadjusted and IPTW-adjusted datasets.

**Table 1.** Clinical and Microbiological Characteristics of Children Who Received Active Versus Inactive Empirical Antimicrobial Therapy for Gram-negative Bloodstream Infection

Clinical and Microbiological Characteristics	Total (N = 659)	Active Empirical Therapy (N = 597)	Inactive Empirical Therapy (N = 62)
**Sex (female)**	274 (41.6%)	255 (42.7%)	19 (30.7%)
**Age**	…	…	…
Neonates (<28 d)	76 (11.5%)	69 (11.6%)	7 (11.3%)
Infants (38–364 d)	151 (22.9%)	141 (23.6%)	10 (16.1%)
Children (1–17 y)	432 (65.6%)	387 (64.8%)	45 (72.6%)
**Acquisition setting**	…	…	…
Community onset	420 (63.7%)	387 (64.8%)	33 (53.2%)
Hospital onset	239 (36.3%)	210 (35.2%)	29 (46.8%)
ICU onset	78 (11.8%)	67 (11.2%)	11 (17.7%)
**Healthcare associated**	440 (66.8%)	391 (65.5%)	49 (79.0%)
**Comorbidities present**	196 (29.7%)	414 (69.4%)	49 (79.0%)
**Types of comorbidities**	…	…	…
Malignancy	244 (37.0%)	220 (36.9%)	24 (38.7%)
Congenital	66 (10.0%)	59 (9.9%)	7 (11.3%)
Gastrointestinal	66 (10.0%)	58 (9.7%)	8 (12.9%)
Hematological	93 (14.1%)	80 (13.4%)	13 (21.0%)
Renal	40 (6.1%)	36 (6.0%)	4 (6.5%)
Prematurity	27 (4.1%)	23 (3.9%)	4 (6.5%)
**Focus of infection**	…	…	…
None identified	227 (34.5%)	200 (33.5%)	27 (43.6%)
Intravascular device	96 (14.6%)	84 (14.1%)	12 (19.4%)
Organ specific	336 (51.0%)	313 (52.4%)	23 (37.1%)
Urinary tract	141 (21.4%)	132 (22.1%)	9 (14.5%)
Intra-abdominal	86 (13.1%)	81 (13.6%)	5 (8.1%)
Mucosal barrier injury	34 (5.2%)	32 (5.4%)	2 (3.2%)
Respiratory tract	10 (1.5%)	8 (1.3%)	2 (3.2%)
Other**^a^**	65 (9.9%)	60 (10.1%)	5 (8%)
**Pathogen**	…	…	…
3GC-resistant Enterobacterales	122 (18.5%)	97 (16.3%)	25 (40.3%)
*Escherichia coli*	231 (35.1%)	220 (36.9%)	11 (17.7%)
*Enterobacter* spp	96 (14.6%)	88 (14.7%)	8 (12.9%)
*Klebsiella* spp	94 (14.3%)	87 (14.6%)	7 (11.3%)
*Salmonella* spp	92 (14.0%)	88 (14.7%)	4 (6.5%)
*Acinetobacter* spp	26 (4.0%)	17 (2.9%)	9 (14.5%)
*Pseudomonas* spp	72 (10.9%)	60 (10.1%)	12 (19.4%)
*Stenotrophomonas maltophilia*	14 (2.2%)	5 (0.8%)	9 (14.5%)
Hard to treat**^b^**	112 (17%)	82 (13.7%)	30 (48.4%)

Abbreviation: ICU, intensive care unit.

^a^Included bone/joint infection, cardiac infection, central nervous system infection, skin and soft tissue infection.

^b^Hard to treat pathogens included *Acinetobacter* spp, *Pseudomonas* spp, *Stenotrophomonas maltophilia*.

### Stratified Analysis

Generalized pairwise comparison approach was also applied to the composite endpoint according to acquisition (non-healthcare vs healthcare-associated), class of Gram-negative bacteria (non-fermenting Gram-negative bacteria, ESBL-Enterobacterales and non-ESBL Enterobacterales) and comorbidity status. Participants were divided into strata, and the pairwise comparison was performed in each stratum. An overall WR for all strata was estimated by combining stratum-specific WRs across the strata and weighted according to numbers in each stratum.

### Statistical Analysis

The χ^2^ test or Fisher exact test was used to compare categorical variables and the Student t test or Mann–Whitney *U* test was used to compare continuous variables. *P* values <.05 were considered statistically significant. Inverse probability of treatment weighting was applied as described by Wang et al [21]. Covariate balance pre- and post-IPTW adjustment were assessed using standardized mean differences (SMDs) and summarized in Supplementary Materials 3. It has been suggested that SMDs less than 0.10 indicate negligible imbalance [22]. Handling of missing data is outlined in Supplementary Materials 4. Unadjusted and adjusted (using stabilized IPTW) win statistics were calculated using the unmatched approach described by Pocock et al [23], Buyse [24], Dong et al [25], and Wang et al [21]. The 95% CIs were derived using the method proposed by Dong et al [26], implemented via the WINS R [27] package. Superiority of AET was defined as a lower bound of the 95% CI for the WR exceeding 1. All analyses were performed in R version 4.2.2 (R Foundation for Statistical Computing, Vienna, Austria) and Stata17 [28].

### Ethical Approval

This study received ethical approval from the Sydney Children's Hospital Network Human Research Ethics Committee (HREC/18/SCHN/72) and local governance approvals at participating sites.

## RESULTS

Six hundred and fifty-nine episodes of GNBSI were included for analysis. Baseline characteristics of the patient cohort are summarized in Table 1. Five hundred and ninety-seven (91%) children received AET, and 62 (9%) received IET. Antibiotics were administered within 2 calendar days of blood culture collection in 588 (98%) AET episodes and 60 (97%) IET episodes, indicating minimal difference in initiation timing between the groups. Only 21 (3.5%) AET episodes received active therapy on the third calendar day. Of those initially receiving IET, 13 (21%) children subsequently received active targeted therapy. Outcomes among episodes classified as receiving IET, stratified by subsequent receipt of active targeted therapy, are presented in Supplementary Materials 5. Median duration of treatment was 10 days (IQR 7–14 days), with no significant difference between AET and IET groups (10 vs 11 days, respectively; *P* = .44). A switch from intravenous to oral therapy occurred in 68/629 (11%) episodes, of which 38 (6%) occurred within 5 days of BSI onset. Intravenous-to-oral switch occurred in 61 (10%) episodes in the AET group and 7 (13%) episodes in the IET group (*P* = .63). Aminoglycosides were the most used antibiotic class in the first 3 days (43% of episodes), followed by piperacillin/tazobactam (38%) and third-generation cephalosporins (38%), noting that percentages exceed 100% as some received multiple antibiotics sequentially or combined. Carbapenems were used as part of empirical therapy in 23% of episodes, with the highest use observed in ESBL-Enterobacterales infections (45%). Children in the AET group were significantly more likely to receive carbapenems (25% vs 6%, *P* < .001) and aminoglycosides (44% vs 28%, *P* = .01) compared with the IET group. Detailed antibiotic use and specific organism data for IET are provided in Supplementary Materials 6.

Eighty-nine patients (14%) experienced at least one event in the composite endpoint: 9 (1%) died within 30 days of BSI onset, 69 (11%) required ICU admission and 17 (3%) had a relapse. Median time to death and duration of hospitalization were 12 days (IQR 4–15 days) and 14 days (IQR 8–31 days), respectively. Table 2 summarizes the components of composite endpoint by treatment group.

**Table 2.** Component Contributions to the Composite Endpoint by Treatment Group

Composite Endpoint Components	Active Empirical Therapy (N = 597)	Inactive Empirical Therapy (N = 62)
30-d ACM	7 (1.2%)	2 (3.2%)
Median time to death within 30 d (days, IQR)	12 (4–22)	8 (1–15)
ICU admission	62 (10.4%)	7 (11.3%)
Clinical relapse	13 (2.2%)	4 (6.5%)
Median duration of hospitalization (days, IQR)	12 (8–21)	13 (6–22)

Abbreviations: ACM, all-cause mortality; ICU, intensive care unit; IQR, interquartile range.

### Inverse Probability of Treatment Weighting

Unweighted SMDs were >0.10 for 10 of 19 baseline covariates, indicating notable imbalance between AET and IET groups. After stabilized inverse probability of treatment weighting, only 2/19 covariates had SMDs >0.10 (Supplementary Materials 3), indicating negligible baseline differences.

### GPC Analyses

**Figure 2.**
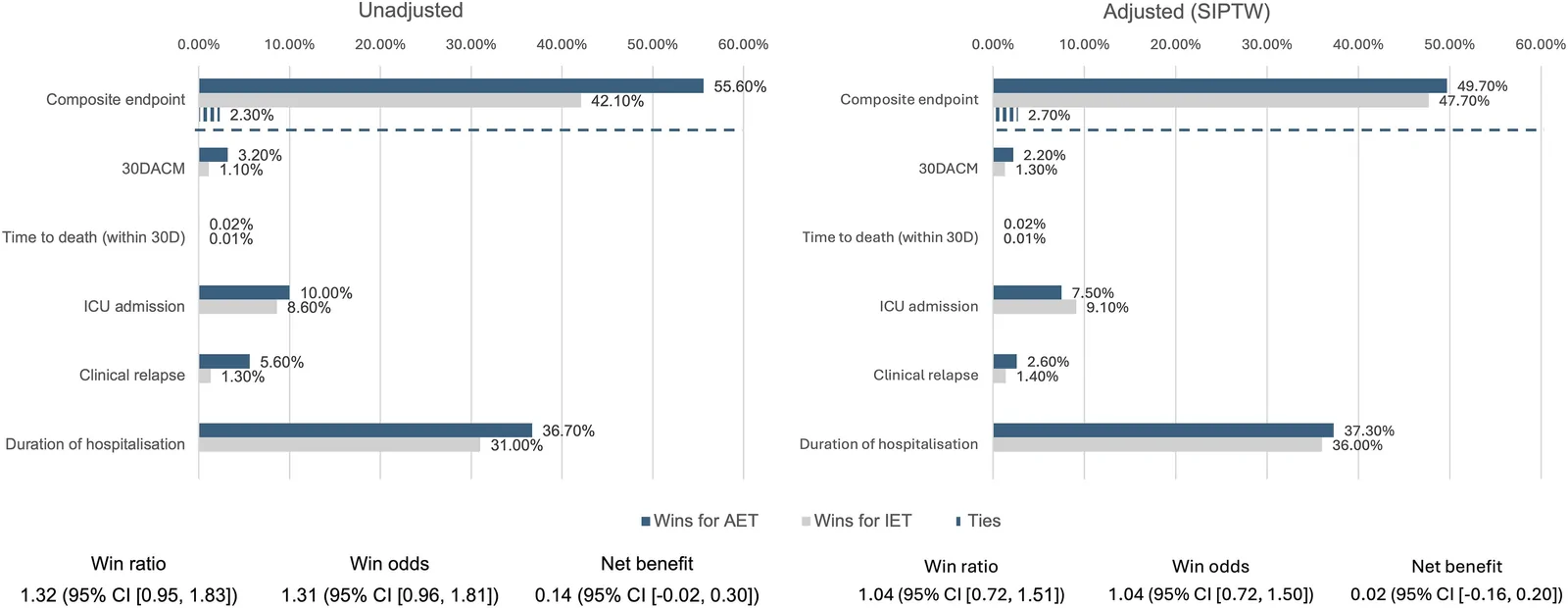
Generalized pairwise comparison analysis of the activity of empirical antibiotic therapy in children with gram-negative bloodstream infection. This figure illustrates the proportion of wins (blue) and losses (orange) from 37 014 paired comparisons of active versus inactive empirical therapy, based on a ranked composite endpoint. A “win’ for 30-day all-cause mortality occurred in 3.2% (unadjusted) and 2.2% (adjusted) of pairs in favor of active therapy, versus 1.1% (unadjusted) and 1.3% (adjusted) in favor of inactive therapy (counted as losses for active therapy). Abbreviations: 30D, 30 d; ACM, all-cause mortality; AET, active empirical therapy; CI, confidence interval; IET, inactive empirical therapy; SIPTW, stabilized inverse probability of treatment weighting.

Using the unmatched approach, each AET patient (N = 597) was paired with an IET patient (N = 62), resulting in 37 014 paired comparisons. The ranked composite analysis showed unadjusted and adjusted WRs of 1.32 (95% CI .95–1.83, *P* = .09) and 1.04 (95% CI .72–1.52, *P* = .83), indicating no significant differences between groups (Figure 2). Events were infrequent across endpoints except for duration of hospitalization, which minimized the number of ties to 2.3% (unadjusted) and 2.7% (adjusted).

Approximately half of the deaths which occurred between 30 and 90 days of BSI onset (10/19 deaths) were assessed to be related to GNBSI. A sensitivity analysis was conducted with 90-day ACM instead of 30-day ACM in the ranked composite analyses, which showed no statistically significant differences between AET and IET, with an unadjusted and adjusted WR of 1.30 (95% CI .93–1.82, *P* = .13) and 0.94 (95% CI .63–1.41, *P* = .76), respectively.

## STRATIFIED ANALYSES

**Figure 3.**
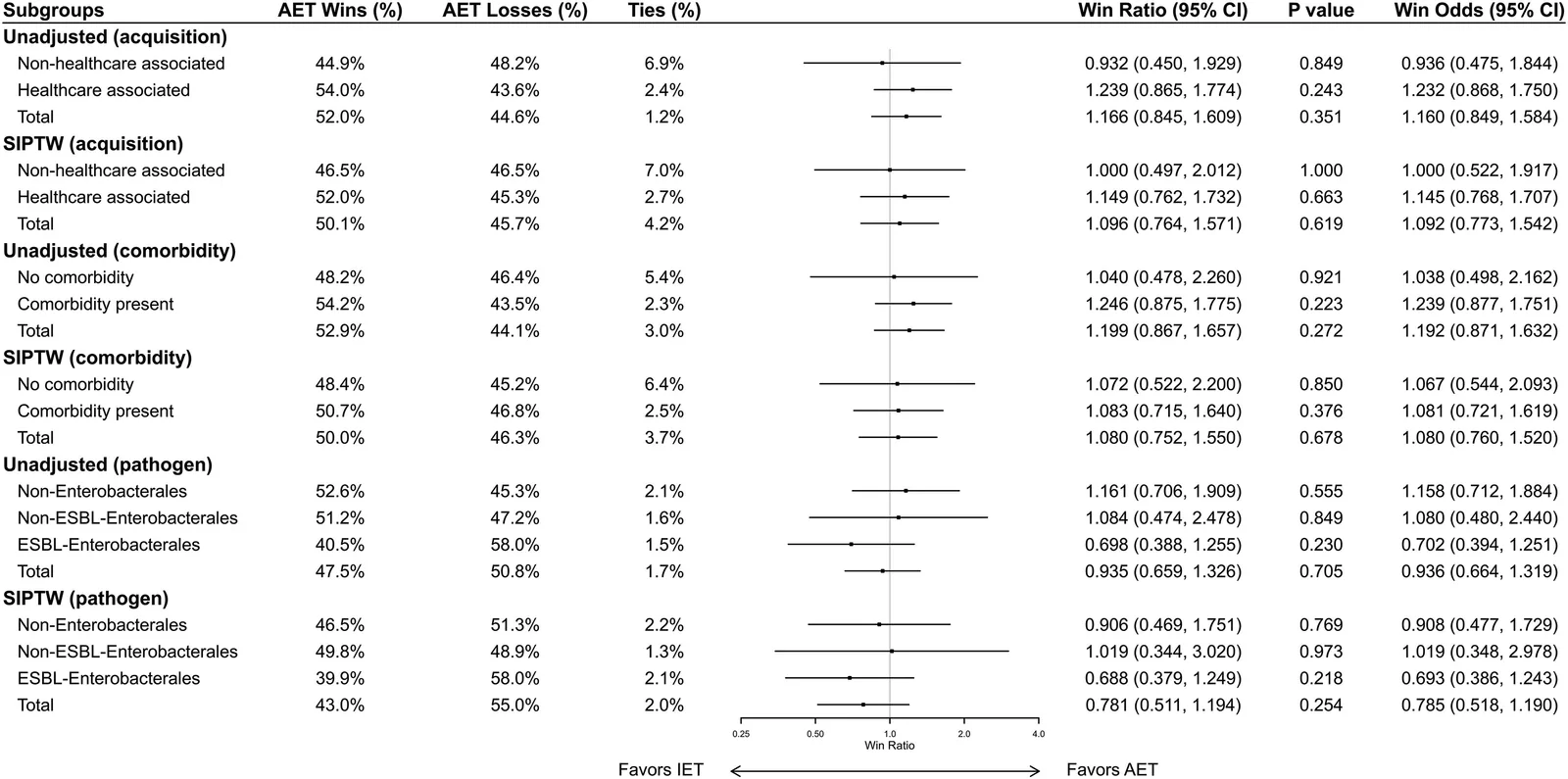
Stratified analysis of the ranked composite endpoint in children with Gram-negative bloodstream infection, before and after stabilized inverse probability of treatment weighting adjustment. The ranked composite endpoint comprised 30-day all-cause mortality, time to death within 30 d, intensive care unit admission, clinical relapse, and duration of hospitalization. Subgroup analyses were conducted by infection setting (healthcare-associated vs non–healthcare-associated), comorbidity status, and pathogen group (non-Enterobacterales, non–ESBL-Enterobacterales, and ESBL-Enterobacterales). Win ratio and win odds estimates, along with 95% CIs and *P* values, are presented for each subgroup and for the overall stratified comparison. Abbreviations: AET, active empirical therapy; CI, confidence interval; ESBL, extended spectrum beta-lactamase; IET, inactive empirical therapy; SIPTW, stabilized inverse probability of treatment weighting.

Figure 3 summarizes the stratified analyses. Win statistics stratified by comorbidity and acquisition were similar to the unstratified estimates. When stratified by pathogen, the unadjusted and adjusted WRs were 0.94 (95% CI .66–1.34, *P* = .71) and 0.78 (95% CI .51–1.19, *P* = .25), respectively. For ESBL-Enterobacterales, the corresponding WRs were 0.70 (95% CI .39–1.26, *P* = .23) and 0.69 (95% CI .38–1.25, *P* = .22), largely reflecting wins in the hospitalization duration endpoint among the IET group (48% vs 28% for AET, Supplementary Materials 7).

## DISCUSSION

This multicenter study evaluated the impact of AET in 659 children with GNBSI using GPC with a ranked composite outcome. Overall, AET within the first 3 days of GNBSI onset was not associated with improved outcomes compared with IET, and stratified analyses by comorbidity and acquisition category similarly showed no significant differences.

These findings challenge the assumption that early AET improves outcomes in serious bacterial infections. Several factors may explain these findings. First, in vitro resistance does not always translate to clinical failure. Agents classified as resistant by MIC breakpoints may still achieve therapeutic concentrations at the infection site, particularly with optimized dosing. This pharmacologic overlap between in vitro resistance and in vivo efficacy may contribute to the modest clinical differentiation observed between AET and IET, especially in high-resource settings such as Australia, where antimicrobial stewardship and dosing optimization are well established.

Clinical context is also critical. This cohort comprised hospitalized children—many with serious comorbidities—who received close monitoring, early escalation of care, and supportive interventions that may have mitigated the impact of antibiotic activity. Thirty-day mortality was low (nine deaths, 1.4%), and resistance among Enterobacterales was relatively low (22% 3GC-resistant), further reducing contrast between groups. The small number of neonates—the subgroup at highest risk of mortality—likely contributed to the low event rate.

Aminoglycosides were the most used antibiotic class for treatment in this cohort. This is consistent with Australian pediatirc empirical antibiotic guidelines [29–31], in which aminoglycosides are frequently used, often in combination with a companion agent that may not necessarily provide gram-negative activity. National surveillance indicates aminoglycoside resistance among pediatric GNBSI isolates in Australia remains relatively low (9.5% gentamicin and/or tobramycin resistance in Enterobacterales) [4], supporting their continued empirical use.

A key limitation is residual confounding by illness severity. Despite IPTW, children who were most unwell may have preferentially received broader empirical therapy, biasing estimates toward poorer outcomes in the AET group (confounding by indication). This possibility likely contributes more to the null association than other measured limitations. Standardized severity-of-illness scores could not be constructed from available data, and severity indicators such as ICU admissions were incorporated within the ranked composite outcome, limiting their suitability for adjustment.

Few pediatric studies have examined AET in BSI [5, 10–12, 32]. Most reported poorer outcomes with IET, typically using 28- or 30-day mortality as the primary endpoint [5, 10–12]. The multinational GARPEC study (N = 452) found higher 30-day mortality among children receiving IET (17% vs 6%; overall 8%) [5]. Conducted across 19 countries with varying resources and resistance rates, its heterogeneity complicates interpretation, but the large, diverse cohort remains an important contribution [5].

In contrast, 2 studies reported no significant differences [12, 32]. A Dutch multicenter study including adults and children with ESBL-Enterobacterales BSI found no association between IET within 24 hours and 30-day mortality, although only 10% were children [12]. Dar et al [32] studied non-critically ill children with sterile-site infections and found that inadequate empirical therapy did not prolong hospitalization; however, because most BSIs in that cohort received AET, inference was limited. Collectively, these studies highlight heterogeneity and the limitations of mortality-based endpoints in children.

Alternatively, clinically distinct sub-phenotypes within pediatric GNBSI may influence both treatment decisions and outcomes. Such sub-phenotypes have been demonstrated in adults with methicillin-susceptible *Staphylococcus aureus* BSI, with differing outcomes [33, 34]. In pediatric GNBSI, possible examples include otherwise healthy children with urinary-source infection who often have simpler courses and may require shorter therapy, compared with children with CVC-associated infections, where comorbidity and source control may drive more complex clinical courses. Similar pediatric GNBSI sub-phenotypes merit exploration in future analyses.

Other limitations include the overall sample size and the small number of IET episodes. Post hoc sample size estimation suggests substantially larger cohorts would be required to reliably detect modest between-group differences and may inform planning of future studies using this approach (Supplementary Materials 8). Further limitations include differences in pathogen mix and prescribing practices (affecting generalizability, particularly in non-tertiary or lower-resource settings), and definitional choices (a three-day empirical therapy window vs 24–48 hours elsewhere [5, 10, 32]; follow-up anchored at culture time). Additionally, antibiotic administration and blood culture timing were recorded by calendar date, introducing some imprecision in defining treatment timing. Detailed data on community antibiotic exposure prior to hospital presentation were unavailable, limiting assessment of its potential influence on treatment outcomes. Immortal-time bias was likely minimal (two deaths within 3 days). Data on source-control interventions, including timing of CVC removal, and pharmacokinetic (PK)/pharmacodynamic (PD) parameters (eg, aminoglycoside dosing, prolonged/continuous infusions) were unavailable and may influence outcomes independent of categorical susceptibility; furthermore, therapy activity in this study was determined using centrally repeated susceptibility testing, whereas clinical decisions relied on local laboratory reporting and routine practice, with variable infectious diseases specialists involvement, which may have resulted in some therapies considered active locally being classified as inactive in the study analysis. Hospitalization duration could not be reliably attributed to GNBSI, and prolonged admissions may sometimes have reflected unrelated clinical needs. Duration was therefore capped at 90 days to reduce the influence of very prolonged hospitalizations. Although hospitalization length was the lowest-priority tier in the ranked endpoint—potentially favoring earlier deaths beyond day 30—a sensitivity analysis yielded similar results.

Key strengths of this study include its large multicenter pediatric cohort, a consumer-informed prespecified ranked composite endpoint aligned with low mortality, and the use of IPTW to reduce confounding. Nevertheless, as with all observational designs, residual confounding remains possible. Future work should incorporate validated severity-of-illness measures, PK/PD data, and cluster-based analyses to identify clinical sub-phenotypes in pediatric GNBSI and evaluate ranked composite endpoints across other infectious syndromes to guide endpoint selection for pediatric BSI trials.

## CONCLUSION

In this large multicenter cohort of children with GNBSI, no significant differences in outcomes were observed between AET and IET after adjustment using a ranked composite endpoint approach. This finding likely reflects low event rates, limited IET exposure, and the multifactorial determinants of clinical recovery. In high-resource settings, the impact of initial empirical activity may be smaller than traditionally assumed, though timely active therapy remains essential in severe illness and in low-resource settings.

## Supplementary Material

ciag234_Supplementary_Data

## Supplementary Data

Supplementary materials are available at *Clinical Infectious Diseases* online. Consisting of data provided by the authors to benefit the reader, the posted materials are not copyedited and are the sole responsibility of the authors, so questions or comments should be addressed to the corresponding author.
