# Supplementary material for: Empirical Antibiotic Activity and Outcomes in Pediatric Gram-negative Bloodstream Infections: A Rank-based Composite Outcome Analysis
Source: Clin Infect Dis. 2026 Apr 9;83(2):e282–90. doi: 10.1093/cid/ciag234 (PMC13537164; doi:10.1093/cid/ciag234)
Supplement: Supplementary file 1 [file ciag234_supplementary_data.docx]

**Supplementary materials**

**S1: Candidate endpoints available for inclusion:**

- All-cause mortality at 7 days
- All-cause mortality at 30 days
- All-cause mortality at 90 days
- Time to death (within X days) - X being the 7, 30 or 90 days.
- Intensive care unit admission
- Clinical relapse with same organism
- Readmission (any cause)
- Total length of hospitalisation from bloodstream infection onset
- Duration of bacteremia (not consistently available, as clearance blood cultures were not obtained in all episodes)

**S2: Covariates used for IPTW analysis**

| **Characteristics** | **Details** |
| --- | --- |
| Age | Neonates <28 days, Infants 28 days – 364 days |
| Sex | Male/Female |
| Acquisition | Community onset or healthcare-associated |
| Selected comorbidities |  |
| Prematurity (if patient is less than 12 months of age) | Yes/No |
| Malignancy | Yes/No |
| Congenital/genetic condition | Yes/No |
| Gastrointestinal condition | Yes/No |
| Haematologic/immunologic condition | Yes/No |
| Renal | Yes/No |
| Presence of CVC | Yes/No |
| Recent surgery (within 30 days of GNBSI) | Yes/No |
| Focus of infection | CVC, organ specific, none |
| Pathogen | *E. coli*, *Klebsiella spp*, *Enterobacter spp*, *Salmonella spp* |
| Hard to treat pathogen (*Acinetobacter spp*, *Pseudomonas spp*, *S. maltophilia*, *Burkholderia* spp.) | Yes/No |

**S3: Standardized mean differences of baseline patient characteristics pre- and post-inverse probability of treatment weighting adjustment**

| **Variable** | **Unadjusted SMD** | **SIPTW-ATE SMD** |
| --- | --- | --- |
| Age (neonates) | 0.01 | 0.15 |
| Age(28d-1yr) | 0.19 | 0.10 |
| Infection source (CVC) | 0.14 | 0.09 |
| Infection source (organ specific) | 0.31 | 0.03 |
| Acquisition (community or healthcare associated) | 0.31 | 0.04 |
| Sex | 0.25 | 0.10 |
| Prematurity | 0.14 | 0.02 |
| Malignancy | 0.04 | 0.12 |
| Congenital | 0.05 | 0.07 |
| Gastro | 0.10 | 0.03 |
| Haematologic | 0.20 | 0.06 |
| Renal | 0.02 | 0.06 |
| CVC | 0.26 | 0.01 |
| Recent surgery (last 30 days) | 0.16 | 0.02 |
| *E. coli* | 0.44 | 0.00 |
| *Klebsiella* spp | 0.10 | 0.00 |
| *Enterobacter* spp | 0.05 | 0.01 |
| *Salmonella* spp | 0.27 | 0.10 |
| Hard to treat pathogens | 0.81 | 0.09 |

SMD = standardized mean difference. SMD <0.1 indicates negligible difference.

SIPTW-ATE = stabilised inverse probability of treatment weighting – average treatment effect.

**S4: Missing data**

1. 35 episodes of non-typhoidal *Salmonella* BSI with missing susceptibility data – given greater than 90% of non-typhoidal *Salmonella* isolates in this cohort were 3GC susceptible, all episodes for which empirical treatment was either 3GC or meropenem were re-coded as having received active empirical antibiotics.
2. 34 episodes with missing duration of hospitalisation from BC onset due to lack of hospital discharge date (identified as “ongoing admission” in the dataset) were censored at 90 days.
3. 1 episode with data missing of duration of hospitalisation from BC onset (due to incorrect discharge date) – a median duration was used (12 days)
4. For those that died (38 episodes), the duration endpoint will be time from blood culture taken to death. If this was longer than 90 days, it was censored at 90 days.
5. 142 episodes with missing data are excluded (missing bacterial species data, antibiotic treatment data, antibiotic susceptibility data or lack of susceptibility interpretative criteria for treatment received).

**S5: Outcomes among episodes receiving inactive empirical therapy stratified by subsequent receipt of active targeted therapy**

| IET (N=62)* | Active targeted therapy (N=13) | Inactive targeted therapy (N=34) |
| --- | --- | --- |
| 30-day all-cause mortality | 0 | 1 |
| ICU admission | 2 | 5 |
| Clinical relapse | 0 | 4 |
| Median duration of hospitalisation (days, IQR) | 16 (12-34) | 12 (6-21) |
| *Incomplete targeted antibiotic data (N=15) | | |

**S6: Antibiotic exposure in the first 3 days following bloodstream infection onset in the inactive empirical therapy group**

| IET (N=62) |  | Bacteria species | | | |  |
| --- | --- | --- | --- | --- | --- | --- |
| Monotherapy | Total | Enterobacterales | *Acinetobacter* spp | *P. aeruginosa* | *S. maltophilia* | Notes |
| AG | 4 | 2 | 1 | 0 | 1 | *E. coli*, ECC |
| Aminopenicillin | 2 | 1 | 1 | 0 | 0 | *E. coli*, *Klebsiella* spp |
| 3GC | 9 | 7 | 1 | 1 | 0 | ECC, *K. aerogenes*, 2x*E. coli*, *K. pneumoniae*, 2x *Salmonella spp* |
| Cefepime | 2 | 1 | 0 | 1 | 0 | *K.pneumoniae* |
| Carbapenem | 2 | 0 | 0 | 0 | 2 |  |
| FQ | 2 | 1 | 0 | 0 | 1 | *E.coli* |
| P/T | 18 | 8 | 3 | 5 | 2 | 3xECC, 2x*E.coli*, 1x*K. oxytoca*, 2x*K. pneumoniae* |
| A/C | 1 | 1 | 0 | 0 | 0 | *K. pneumoniae* |
| Total | 40 | 21 | 6 | 7 | 6 | 40 |
|  |  |  |  |  |  |  |
| Sequential antibiotics | Total | Enterobacterales | *Acinetobacter* spp | *P. aeruginosa* | *S. maltophilia* | Notes |
| Cefazolin to FQ | 1 | 0 | 0 | 0 | 0 | *Campylobacter spp* |
| 3GC to P/T | 1 | 0 | 0 | 0 | 1 |  |
| 3GC to aminopenicillin | 1 | 0 | 1 | 0 | 0 |  |
| AG to carbapenem to FQ | 1 | 1 | 0 | 0 | 0 | *Enterobacter spp* |
| Total | 4 | 1 | 1 | 0 | 1 |  |
|  |  |  |  |  |  |  |
| Combination antibiotics | Total | Enterobacterales | *Acinetobacter* spp | *P. aeruginosa* | S. maltophilia | Notes |
| AG & 3GC | 3 | 1 | 0 | 0 | 2 | Serratia spp |
| 3GC & Azithromycin | 2 | 2 | 0 | 0 | 0 | 2x*Salmonella spp* |
| P/T & AG | 4 | 0 | 1 | 3 | 0 |  |
| Aminopenicillin & 3GC | 3 | 3 | 0 | 0 | 0 | 3x*E. coli* |
| Aminopenicillin & AG & 3GC | 1 | 1 | 0 | 0 | 0 | *E.coli* |
| Aminopenicillin & AG | 1 | 0 | 0 | 1 | 0 |  |
| C/T & AG | 1 | 0 | 0 | 1 | 0 |  |
| Cefepime & AG | 1 | 1 | 0 | 0 | 0 | ECC |
| AG & carbapenem | 1 | 1 | 0 | 0 | 0 | *K. pneumoniae* |
| Total | 17 | 9 | 1 | 5 | 2 | 17 |
|  |  |  |  |  |  |  |
| Combination to sequential antibiotics | Total | Enterobacterales | *Acinetobacter* spp | *P. aeruginosa* | *S. maltophilia* | Notes |
| 3GC & AG to P/T | 1 | 0 | 0 | 0 | 1 |  |

A/C = amoxycillin/clavulanic acid, AG = aminoglycoside, C/T = ceftolozane/tazobactam, ECC = Enterobacter cloacae complex, FQ = fluoroquinolone, IET = inactive empirical therapy, P/T = piperacillin/tazobactam, 3GC = third generation cephalosporin.

Of those that received IET but active targeted therapy: 1x *Acinetobacter* spp, 2x ECC, 1x *K. aerogenes*, 1x *E. coli*, 2x *Klebsiella* spp, 4x *P. aeruginosa*, 2x *S. maltophilia*

**S7: Stratified analyses based on pathogen**

| **ESBL-Enterobacterales stratified analysis (unadjusted)** | **AET wins** | **IET wins** |
| --- | --- | --- |
| 30D ACM | 3.88% | 2.97% |
| Time to death (within 30D) | 0.12% | 0% |
| ICU admission | 7.09% | 7.26% |
| Clinical relapse | 3.55% | 1.91% |
| Duration of hospitalisation | 25.90% | 47.80% |

| **ESBL-Enterobacterales stratified analysis (SIPTW)** | **AET wins** | **IET wins** |
| --- | --- | --- |
| 30D ACM | 2.33% | 2.98% |
| Time to death (within 30D) | 0.07% | 0% |
| ICU admission | 7.66% | 7.36% |
| Clinical relapse | 1.73% | 1.92% |
| Duration of hospitalisation | 28.10% | 47.70% |

ACM = all-cause mortality, AET = active empirical therapy, IET = inactive empirical therapy, ICU = intensive care unit, SIPTW = stabilized inverse probability of treatment weighting, 30D = 30-day

**S8 Post hoc sample size calculation**

Sample size calculations for generalized pairwise comparisons analyses can be estimated in Stata from the expected distribution of each outcome in the hierarchy. The sample size calculations are based on the formula of Yu and Ganju. The Stata software assumes a fixed trial duration and the components at each level are independent of one another.

The sample size requirements to achieve 80% power to demonstrate a superior effect of active over inactive empirical therapy with a two-sided alpha level of 0.05 and a 10:1 treatment allocation using the distribution of outcomes observed in our study would require the following:

References:

Collier T, Gregson J. WINRATIOTEST: Stata module to calculate the unmatched Win Ratio for prioritised outcomes [Available from: <https://ideas.repec.org/c/boc/bocode/s458984.html>.

Yu RX, Ganju J. Sample size formula for a win ratio endpoint. Statistics in Medicine. 2022;41(6):950–63.

Pocock SJ, Ferreira JP, Collier TJ, Angermann CE, Biegus J, Collins SP, et al. The win ratio method in heart failure trials: lessons learnt from EMPULSE. European Journal of Heart Failure. 2023;25(5):632–41.
